# The Coming of the Mob

**Published:** 1921-03-26

**Authors:** 


### "The Coming of the Mob."

In our last issue appeared a vigorous and sincere protest from "Ierne" against the proposed admission to the Irish Register of existing nurses whose qualification rests on one year's hospital or Poor-law infirmary training. It is likely that it voices the feelings of many nurses, both in Ireland and in other parts of the United Kingdom, for although registration schemes have always involved the recognition of the "existing" nurse, it is quite certain that nurses as a body have never realised that this was so.

"Ierne" describes the women who are to be permitted to register during the two years of grace as a "mob of untrained nurses." In this we think he does little justice to the very large body of women whose qualifications would not suffice to secure them registration on their own merits. We all know and appreciate many nurses, whom we prefer to call partially trained rather than untrained. Many of them are as the salt of the earth. They have missed the higher training now regarded as essential to proficiency by the misfortune of entering institutions not adapted for giving full training, or by other circumstances. They suffer from the long continuance of a bad system, and at any rate it may be claimed that registration from now on will prevent this wilful sacrifice of potential trained nurses for lack of a central nursing educational system.

But, take them at their worst—and it must be admitted that the partially trained are a very mixed lot—what registration does for them is to bring them within the reach of discipline. It is often forgotten that unregistered nurses are a law unto themselves. They cannot be deprived of the name of nurse, whatever they may do or leave undone. As soon, however, as their names are inscribed on the Register they become amenable to the General Nursing Council. Should they present false credentials, attempt to impose on the public, become guilty of professional malpractice, they can be brought to book and their names removed from the Register. It is pretty certain that women of this type will desire nothing less than to be brought into the fold. Their stronghold is the present chaos in the nursing profession. The useful existing nurse will go on practising, cheered by admission to the Register, and the public cannot, in fact, do without her. The pseudo-nurse will prefer the shadows of irresponsibility.

We are certain that "Ierne" is mistaken in surmising that "neither the public nor the profession will be able to discriminate" between fully trained and partially trained registered nurses after a few months have passed. The Army, the Navy, and other public services will, of course, require all their candidates to be registered nurses. But no service could admit candidates solely because they complied with this stipulation. No employers of nurses will in any degree alter their standard or abate one jot of their prescribed qualifications. Why should they? Registration will provide them with a very important measure of protection, for the credentials of all candidates will come before the employer stamped with the hall-mark of authenticity. Shuffling and prevarication, plausible stories of the theft and loss by fire or shipwreck of certificates and testimonials will have been sifted by the properly constituted authority, and the registered nurse will be proved to be what she professes to be—either partially trained with satisfactory references, or fully trained and certificated. Will this be no gain to the profession?

It is still further urged that the partially trained may impinge on the practice of their better equipped sisters, say, in nursing homes or with busy doctors, only too thankful to take it for granted that a registered nurse is all right. It is possible. But is the nursing world free at this moment from this abuse, under different forms? Notoriously the partially trained can insinuate themselves into almost every sphere of work. But whereas at present they can cast a glamour over their past doings, through which it is difficult to see very clearly, after registration they will be pinned down to facts. The doctor, the superintendent, the nursing board imposed on in the future when engaging nurses will be beguiled without the shadow of an excuse. The *bona-fide* woman will be discriminated as clearly as possible by the printed record in the Register from the fully certificated medical and surgical nurse.

The experience of the Central Midwives Board goes to show that registration, so far from lowering the status of highly trained women, tends to put them quite in a different category and lift them from all possibility of confusion with the women who have had little or no training. To have excluded *bona-fide* midwives from registration would have been not merely a great injustice, but a national misfortune. By twos and threes, continuously, watchfully, the mischievous have been eliminated, the better sort instructed, and the standard of training raised. It is our earnest conviction that the same result will follow the registration of nurses.

